# FINANCIAL EXPLOITATION AND MENTAL HEALTH OF HOLOCAUST SURVIVORS: THE MODERATING ROLE OF POSTTRAUMATIC SYMPTOMS

**DOI:** 10.1093/geroni/igad104.0143

**Published:** 2023-12-21

**Authors:** Gali Weissberger, Moshe Bensimon, Amit Shrira

**Affiliations:** Bar Ilan University, Ramat Gan, HaMerkaz, Israel; Bar-Ilan University, Ramat Gan, Tel Aviv, Israel; Bar Ilan University, Ramat Gan, HaMerkaz, Israel

## Abstract

Financial exploitation (FE) is associated with devastating emotional, health, and economic consequences. We examined whether anxiety and depressive symptoms associated with self-reported history of financial exploitation (FE) are more pronounced amongst Holocaust survivors, especially those with high-level posttraumatic stress disorder (PTSD) symptoms. A community-based cohort of 137 Israeli older adults born prior to 1945 were included in the study sample. Holocaust survivors (n = 61) were participants who reported living in a European country occupied or dominated by Nazi or pro-Nazi regimes between 1939-1945. Groups were further subdivided into survivors with low or high levels of PTSD symptoms (≥31 on the PTSD Checklist; PCL-5). Questionnaires assessed FE history, posttraumatic symptoms (PCL-5), depressive symptoms (PHQ-9), and anxiety (GAD-7). Age, education, self-rated health, and non-Holocaust lifetime adversity were also measured and included as covariates. Hierarchical linear regression models revealed that relationships between FE and depressive and anxiety symptoms were significant only among survivors (p = 0.005 and p = 0.008, respectively). The interaction between PTSD symptom level group and FE was also significant for both depressive (p = 0.007) and anxiety (p = 0.012) symptoms, such that survivors with PTSD who reported FE had significantly greater symptoms of depression and anxiety compared to all other groups. Findings suggest that the experience of FE may be particularly impactful amongst survivors who continue to struggle with posttraumatic symptoms related to the Holocaust. Future studies may consider examining whether findings are relevant to other groups with PTSD.

